# Computational mechanism of postponed decisions

**DOI:** 10.1186/1471-2202-12-S1-P213

**Published:** 2011-07-18

**Authors:** Marina Martinez-Garcia, Gustavo Deco, Edmund T Rolls, Ranulfo Romo

**Affiliations:** 1 Department of Technology, Universitat Pompeu Fabra, 08018, Spain; 2 Oxford Centre for Computational Neuroscience, Oxford, UK; 3 Instituto de Fisiología Celular-Neurociencias, Universidad Nacional Autónoma de México, México D.F.,Mexico

## 

The perceptual decision making process may involve a comparison between several stimuli occurring either simultaneously or successively. Depending on environmental demands, the response is given immediately or is delayed. A well-known paradigm for research in decision making is the comparison of two vibrotactile stimuli (f1, f2) applied to the fingertips with a fixed delay period between them. To complete this cognitive task the subject needs to store the information about the first stimulus, f1, in working memory and to compare it with the second one, f2, to make a decision of whether f1<f2 or f1>f2. Further he needs to store in working memory its appropriate response. Along those lines, Lemus et al. (2) introduced an additional delay between the second stimulus and the subject's response. This new operation requires that the subject stores the somatosensory information, the decision and/or the response in working memory during this second delay period. We analysed neurons recorded from the Medial Premotor Cortex in monkeys during the above task. We found some neurons encoded the response just in two periods: after the second stimulus (f2) and when the answer is required, 3 s later. Moreover, those neurons only show relevant activity in these periods.

In the present work we investigated the mechanisms that allow the neurons to encode the response information. For this purpose we use Mutual Information (MI) analysis in order to decode the information carried by the firing rates. We measured the MI between the following two variables: the firing rates and the behavioral response (f1<f2 or f1>f2) made at the end of the trial. We found that for 157 neurons (18.1% of 867), the MI becomes significant during the second stimulus (f2), it disappears during the post-f2 period, and becomes significant again at the time of the response. Our hypothesis is that this particular pattern is due to a combination of signal-to-noise ratio in the rates, which makes it impossible to decode the information contained in the rates in the postponed response delay period after f2.

We hypothesize that the neurons can use synaptic facilitation to allows them to hold the information during the delayed response memory period. Here, we present a biophysically realistic model for decision making with synaptic facilitation (3) extended to account for the activity in the delayed response period. We use integrate-and-fire neurons with three types of receptors mediating the synaptic currents: the excitatory recurrent postsynaptic currents are mediated by AMPA (fast) and NMDA (slow) receptors and the inhibitory recurrent postsynaptic currents to both excitatory and inhibitory neurons are mediated by GABA receptors. The external excitatory recurrent postsynaptic currents injected onto the network are driven only by AMPA receptors.

The results show that the network is able to model this neuronal activity using a synaptic facilitation mechanism. After a decision making period the network is able to remember the response despite a low firing level in the delayed response period and to retrieve the memory evident in a high firing rate when a non-specific stimulus is provided just before the response is required. Thus a synaptic facilitation mechanism can implement a mechanism for postponed decisions which can be correct even when there is little neuronal firing during the delay period before the postponed decision.
